# Radiation: Tanning Trippers Get UV High

**Published:** 2006-07

**Authors:** Adrian Burton

It has long been suspected that cutaneous endorphins are produced during exposure to UV light. Now research published in the April 2006 issue of the *Journal of the American Academy of Dermatology* suggests that frequent users of tanning beds may become addicted to these endorphins. Moreover, blocking the effects of the endorphins could lead to withdrawal symptoms.

“This might explain why some people appear to be hooked on sunbathing and why frequent users of tanning beds say they experience a positive mood change or are more relaxed after a session,” says coauthor Steven Feldman, a professor of dermatology at Wake Forest University School of Medicine.

Feldman’s team thought that blocking this endorphin rush might cause such people to lose some of their tanning enthusiasm; what they didn’t expect was for some to develop withdrawal symptoms.

The subjects included eight frequent tanners (who used tanning beds 8 to 15 times per month) and eight infrequent tanners (who used them up to 12 times per year). The researchers administered either a placebo or 5, 15, or 25 mg of naltrexone, a central and peripheral opioid receptor blocker; this blockage causes withdrawal symptoms in opioid drug–addicted people but not in nonaddicted people. The subjects were then asked to lie for 10 minutes on each of two tanning beds, one a true UV bed, the other rigged not to deliver UV light. Afterwards, the subjects, who were blind to the test conditions, were asked to describe which session made them feel best.

With the placebo and the 5-mg naltrexone dose, the frequent tanners showed a clear preference for the UV bed—and more strongly so than the infrequent tanners. But this preference fell away with the 15- and 25-mg doses of naltrexone, “suggesting that light-induced endorphins are reinforcing [frequent tanners’] behavior,” says report coauthor Mandeep Kaur, also a dermatology professor at Wake Forest University School of Medicine.

Further evidence of this was seen when half of the frequent tanners developed nausea and jitteriness with the 15-mg dose. “These are common [opioid drug] withdrawal symptoms,” explains Feldman, “and they were bad enough for two subjects to drop out.” Although there were no further problems at the 25-mg dose, Feldman says these results suggest that frequent tanners suffer some degree of dependency on endorphins.

“Clearly tanning is not as addictive as smoking,” remarks Robert Dellavalle, an associate professor of dermatology at the University of Colorado Health Sciences Center. “Just look at the prevalence of smoking in middle age—twenty percent in the UK and the United States. In contrast, there is a steep drop-off in the prevalence of tanning as people age.”

Still, says, Feldman, although it’s not time for the Drug Enforcement Administration to raid beauty parlors, “these results do raise questions about the safe use of tanning beds.”

## Figures and Tables

**Figure:**
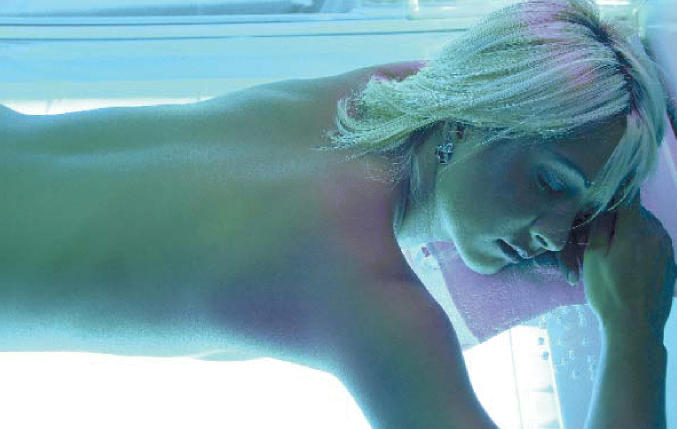
Sun-sations A new study shows why tanning bed rays feels so good to some people: UV light produces endorphins to which frequent tanners may become addicted.

